# 

**Published:** 1895-02

**Authors:** 


### CONTENTS.

ORIGINAL ARTICLES—

The Element of Causation in Abdominal Contusions. By Thomas H. Manley, M. D., New York .......................... 65

SELECTIONS AND ABSTRACTS—

Puerperal Infection of the Urinary Tract and of the Rectum. By Richard C. Norris, M. D., Philadelphia .................... 72

A Case of Epidemic Cerebro-Spinal Meningitis. By W. Jarvis Barlow, M. D., New York .................................. 80

A Case of Carcinoma of the Parturient Uterus, Removed Three Days After Confinement—Recovery. By George H. Noble, M. D., Atlanta, Ga. .......................................... 83

"Stammering of the Urinary Organs" (Paget). By J. Henry C. Simes, Philadelphia ........................................ 88

Acquired Idiosyncrasy for Quinine, Showing Peculiar Cutaneous Manifestations. By Charles W. Allen, M. D., New York ..... 94

SOCIETY REPORTS—

Richmond (Va.) Academy of Medicine and Surgery.................. 104
Transactions of the College of Physicians of Philadelphia. By William J. Taylor, M. D........................................ 109
Philadelphia Academy of Surgery........................................ 111
President's Address Before Atlanta Medical Society. By C. D. Hurt, M. D........................................................ 113

BOOK REVIEWS—

A Manual of Surgery—General and Operative. By John Chalmers Dacosta, M. D., Philadelphia........................ 115
International Clinics—A Quarterly of Clinical Lectures on Medicine, Etc. Edited by Judson Daland, M. D............... 116

EDITORIAL—

Editorial Notes.................................................... 116
Special Notes...................................................... 122

